# Review of Medical Malpractice Issues in Malaysia under Tort Litigation System

**DOI:** 10.5539/gjhs.v6n4p76

**Published:** 2014-04-07

**Authors:** Siti Naaishah Hambali, Solmaz Khodapanahandeh

**Affiliations:** 1 National University of Malaysia (UKM), Bangi, Selangor, Malaysia

**Keywords:** medical malpractice, Tort, Malaysia

## Abstract

Medical malpractice cases are a matter of much concern in many countries including Malaysia where several cases caught the attention of the public and authorities. Although comprehensive annual statistics on medical negligence claims are not available in Malaysia since such data are not collected systematically in this country there are indications of an upward trend. Medical malpractice cases have been publicized by the media, academic researchers and in government annual reports prompting government policy makers, oversight agencies and the medical profession itself to take appropriate action. The increasing dissatisfaction with the current tort litigation system requires exploring alternatives and new approaches for handling medical malpractice cases. This study aims to examine the difficulties inherent in the tort system in Malaysia for solving medical malpractice claims and evaluates the structure of this system from the perspective of effectiveness, fairness, compensation, accessibility, and accountability.

## 1. Introduction

Medical malpractice has become very serious matter recently as the number of claims is on the rise in many countries. Empirical studies have noted significantly high rates of medical malpractice.

The Congressional Budget Office (CBO) noted 181,000 severe injuries attributable to medical negligence in 2003. Data for 2008 studied by Health Grades, the leading healthcare rating organization in the US, found that Medicare patients who experienced a patient-safety incident had a 20% chance of dying as a result.

The figures from the NHSLA’s Annual Report and Accounts 2010, 2011 reveal that, there is an increase in claim numbers. The number of clinical negligence claims reported to the NHSLA in 2009-10 was 6,652, which represents a 10 percent increase over 2008-09 (6,088) which, in turn, recorded an 11 percent increase over 2007-08 (5,470). Again the number of clinical negligence claims reported in 2010/11 was 8,655, which represents a 30 percent increase over 2009/10 (6,652). Although comprehensive annual statistics on medical negligence claims are not available in Malaysia since such data are not collected systematically in this country there are indications of an upward trend. Ministry of Health statistics reveal that between 1986 and 1990, 61 medical negligence claims were made against government physicians, averaging about 12 claims per year. The figures stood at 20 and 16 in 1991 and 1992 respectively. It is believed that many more cases are actually settled out of court (Tay Pek, 1998). Radhakrishnan, the Malaysian Medical Protection Society’s legal adviser said, “Although official statistics on the exact number of medical accidents in clinics and hospitals were not available, the increase could be seen from the rise in premiums paid by doctors for protection against malpractice suits” (Radhakrishnan, 2003). Also Milton Lum, The Medical Defence Malaysia (MDM) board member said, based on the increase in indemnity subscriptions, there seems to be an increase in the number of litigations against doctors. He further added Health Ministry data showed an increase from 29 to 56 cases against doctors from 2006 to 2011 (Chin, 2013).

According to Civil Division of Attorney General’s Chambers, the average number of medical negligence cases in period of five years from 2007-2011 has been risen by 46.8%, comparing to 2002-2006 with average of 33.6 (Attorney General’s Chambers, Civil Division, Bi-Annual Report 2010 – 2011). Similarly the Medico Legal Section of Ministry of Health revealed that, the number of complaints received by the Section showed an increasing trend from year 2006, with 1394 complaints received in 2011 (MOH Annual Report, 2011).

The issue is not only the increasing number of medical malpractice claims across the world but also inability of tort system to deliver justice through fair and adequate compensation. Generally the tort system has been known as inefficient system for delivering compensation as well as non-legal remedy such as explanation and apology (Khodapanahandeh & Hambali, 2013). Compensation is often unpredictable and success may not be due to the merits of the claims. These unpredictable outcomes of tort litigation are the result of the availability and dependability of evidences and witnesses, the quality and expertise of legal representation, the financing of the litigation, the attitude of the judges, and many more (Kassim & Najid, 2013).

Essentially, the system is slow, consuming four years on average to resolve malpractice issues (Cohen & Hughes, 2007), costly (Studdert et al., 2006), inefficient and unrealistically difficult for many injured victims (McLean, 1988). Rehabilitation is also hampered by the prolonged adversarial system (Kassim, 2003). It is often difficult for a plaintiff to succeed in medical negligence claims while logistic issues are fairly unsurmountable for proceeding and continuing with any action (Davies, 1998). This discourages many potential litigants from pursuing their claims, and cases that are pursued are unrepresentative of the number of medical errors occurring while of claims made only a small proportion received compensation. According to studies by Localio et al. (1991) and Mello et al. (2006), under best circumstances, negligence file claims resulted in a mere 2% to 3% of awards for injuries and only half of applicants received financial compensation. Often, litigation is balanced incongruously with the value of the claim where no award was made in 25% of cases that merited compensation.

An increase in the number of claims from medical malpractices would involve huge costs in verifying complaints making it all the more important to study and improve existing related laws. The increase must lead to greater attention and radical changes to health care systems (Khodapanahandeh, 2013). Otherwise Malaysia will be faced with a sharp growth in complaints against physicians as experienced in countries such as the United States, Australia, Canada, and the United Kingdom.

Medical malpractice issues have been investigated from a variety of perspectives in the literature. They include the rise in medical malpractice claims, failure of the system in delivering accountability, and the inaccessibility of the litigation system due to obstacles involved such as the lengthy litigation period, high costs, limitation period, inaccessibility to medical records and difficulties in obtaining opinions of expert medical witnesses. The study examines the difficulties inherent in the tort system in Malaysia in settling medical malpractice claims and evaluates the structure of this system from the aspect of effectiveness, compensation, fairness, accessibility and accountability. The paper is based on library research. The data for this research is collected electronically (Medline, PubMed, and Google Scholar) and manually from various papers, journals, researches and books using key words and phrases such as Medical Malpractice, Tort System and Malaysia to obtain public policy studies, case analyses, law review articles and healthcare analysis articles.

## 2. Discussion

### 2.1 Difficulties in Pursuing Medical Malpractice Claims

From a plaintiff’s perspective, any compensation received in medical malpractice cases are offset by the cost and lengthy time period involved in bringing the suit to court. In addition, the number of claims successfully adjudicated demonstrates the weakness of the current system to adequately compensate an injured victim. In England, a report on civil liability and compensation for personal injury stated that “the proportion of successful claims for damages in [court] is much lower for medical negligence than for all negligence cases” (Royal Commission on Civil Liability and Personal Injury, 1978). As compensation depends upon successful action, the present system leaves many victims uncompensated for injuries. From the perspective of the defendants the increasing number of claims forces physicians to pay higher annual insurance premiums as protection against medical malpractice suits. They also believe that damage awards have no deterrent effect on medical malpractice; instead the higher medical malpractice premiums drive many physicians away from particular specialties and services.

#### 2.1.1 The Lengthy Period and Costly Nature of Pursuing a Claim

The tort compensation system has been known to be costly. High administrative costs are due to the nature of the two principal criteria for compensation, namely, case-by-case determination of fault and lump-sum findings of damages under uncertain guidelines. The main contributor to the high costs in the tort system is the delay involved in the pursuit of a claim (Kassim, 2009). According to the closed-claims study from the United States General Accounting Office, the typical physician claim took about 14 months to be filed: the low was under one month, the high, over 18 years (GAO 1984). According to Jury Verdict Research, average litigation time for medical malpractice in 2000 was 45 months as measured from incident date to trial date (Jury Verdict Research, 2001). In Malaysia the entire litigation process for medical negligence requires an average of about a minimum of 15 years, and may take up to 25 years, from date of injury to the conclusion of the case (Foo Fio Na v Hospital Assunta &Anor [^1999^] 6MLJ). An instance of this is the case of Dr Chin Yoon Hiap v Ng Eu Khoon & Ors [^1998^] where litigation was initiated on 1981 and judgment finally delivered in 1997.

To cover the high cost of litigation, both the Government and the Bar Council provide legal aid in Malaysia. Although the aid is provided it is rarely availed as many complainants are excluded from the system because they do not qualify financially or are too poor to fund an action unaided. To qualify for legal aid an application has to satisfy the “means test” (Legal Aid Act 1971) imposed by the Legal Aid Act, 1971 failing which the patient is left without the financial resources to obtain the necessary legal representation. Compounding this is the fact that the provision for legal aid by the government is small forming only 0.001% of Malaysia’s annual GDP in 2009 (Nekoo, 2009) and extremely low when compared to the figures in developed countries. According to the Council of Europe, public budgets allocated for legal aid per inhabitant in 2010 was 0.29% for Northern Ireland, 0.21% for UK-England and Wales, and 0.05% for Sweden (CEPEJ 2012 Report). Other countries like Canada, New Zealand and Australia assigned 0.05%, 0.07% and 0.04% respectively to legal aid expenditure per head as a proportion of GDP in 2008/2009 (“International Comparisons of Public Expenditure on Legally Aided Services, Ad hoc Statistics Note Ministry of Justice,” 8 September 2011). Even compared to other Asian countries like Taiwan and Hong Kong which allocated approximately 0.05% of their GDP for legal aid, Malaysia fared badly (Nekoo, 2009). It is apparent that the Government of Malaysia could do better in serving the needs of those who require legal assistance but are unable to afford it.

#### 2.1.2 Limitation Period

Medical negligence claims are time-bound and have to be initiated within a specified period. This is known as the limitation period and covered under the Malaysia Limitation Act 1953(Act 254). As noted by McHugh, a former justice of the High Court of Australia, the rationale behind the limitation period is to protect defendants from stale claims and encourage plaintiffs to proceed expeditiously. This will provide finality and provide confidence to sued parties that potential claims are barred after a certain period of time. Imposing limitation periods on actions also prevent loss of relevant evidence with the passage of time. (Brisbane Authority v Taylor (1996) 70 ALJR 866 at 871-2).

Under the law of torts claims cannot be brought after a lapse of six years from the date on which the cause of action occurred(Limitation Act 1953). In torts actionable per se, such as trespass to the person, the cause of action normally accrues at the date of defendant’s wrong, whereas with tort actionable its only on proof of damage, such as negligence, the action accrues when the damage occurs (Kassim, 2008) and this is usually not the same time with the defendant’s breach of duty but later. For example, in cases of anesthetic and surgical mishaps, damage will flow immediately from the negligent act but where prescription of a drug is involved, which cause a delayed kind of harm, damage may only occur years after it is used (Nelson-Jones, 1995). In certain cases, the cause of action may have already expired by the time the victim discovers the potential harm from the drug prescription.

Unfortunately, the Malaysia Limitation Act 1953 does not provide for such a situation as caused by latent injuries whether with regard to personal injuries or any other damage caused by negligence. This situation was resolved in the UK through the enactment of section 11 (4) of the Limitation Act 1980 which allows time to run from the date of knowledge of existence of the cause of action. The injury is considered significant “if the person whose date of knowledge is in question would reasonably have considered it sufficiently serious to justify his instituting proceedings for damages against defendant who did not dispute liability and was able satisfy a judgment”(Limitation Act 1980 UK section 4(2)). This is especially important in medical negligence claims where expert medical opinions are required that there may be liability, and according to these provisions the limitation period does not run until the plaintiff secures such advice.

Also New Zealand solved this issue by Limitation Act 2010, which replaced to 1950 Act. Under the 2010 Act, if the plaintiff is claiming a sum of money, he must bring the claim within 6 years of the event his claim is based on. The time period may be extended if, for example, he didn’t know about the event in question in the initial 6-year period or if he was injured during that period. Court is able to order that no limitation period applies in cases of abuse and personal injury caused by gradual process, disease or infection. Since no such provisions exist under the Malaysia Limitation Act 1953 the problems caused by latent injuries or by medical negligence cannot be solved. Thus provisions such as section 11 (4) of the English Limitation Act 1980 or New Zealand Limitation Act 2010 should be inserted into Malaysian Limitation Act 1953 to address existing inadequacies.

#### 2.1.3 Access to Health Records

Among the procedural obstacles faced by patients in making claims for medical negligence is obtaining the relevant medical records without having to initiate court action. To prepare a medical malpractice case the parties need to seek a specialist in the particular field, obtain medical records from all sources, and the advice of experts and counsels. In many jurisdictions, simplified procedures allow parties to obtain hospital records such as in the UK where such access is provided under various statutory provisions such as the Data Protection Act 1984, Access to Health Records Act 1990, and Access to Medical Report Act 1988 and the Supreme Court Practice 1997. These allow patients not only to ensure that records are in accurate form but are also relevant in the context of litigation as a means of establishing any errors and oversights during treatment. In the United States many states have enacted legislation to ensure access to health records in both the public and private sectors while in New Zealand, the Health Information Privacy Code, which came into force in 1993, provides for an enforceable right of access to medical records.

In Malaysia, whether an action of medical negligence is to be initiated will depend on the amount of information a patient is able to provide and the medical complexity of the treatment. It is impossible for a patient even to go about beginning to make a claim against his doctor unless he can provide some particulars of misconduct. All too often such particulars can only be secured if the patient has access to his own medical records. According to the Guideline of the Malaysian Medical Council, “A patient’s medical record is the property of the medical practitioner and the healthcare facility and services which hold all rights associated with ownership. They are also the intellectual property of the medical practitioner who has written them, and also belong morally and ethically to the practitioner and the patient” (MMC Guideline 002/2006). Unfortunately medical practitioners and hospitals are usually reluctant to produce the records if they suspect they will be used to establish errors or malpractice. In principle there is no reason to deny a patient his medical records, but as the law currently stands only the courts can compel access. In Toh Kong Joo v Penguasa Perubatan Hospital Sultanah Aminah, Johor Baru ([^1990^]2 MLJ 235) the applicant had to resort to a court proceeding before succeeding in obtaining a copy of his medical report. Alternatively, to avoid separate court proceedings the patient may be forced to issue a generally endorsed writing, and then seek inspection of the hospital records by way of discovery under relevant rules of the court (Order 24, r.10 of Rules of Court 2012).

The only choice open for obtaining medical records is through a pre-trial discovery which commences after pleading. This procedure is governed by Order 24 of the Rules of Court 2012. The new Rules, which came into effect on 1 August 2012, replaced the previous Rules of High Court 1980 and deleted Order 24, r. 1 & 2 of the Rules of 1980 related to discovery of documents by parties without an order. According to the new rules there is no more mutual discovery of documents and discovery by parties without an order from the court.

Unfortunately the new rules not only did not extend discovery of documents in the pre-action stage but removed the mutual or automatic discovery by consent of parties. Although Order 34, which is known as a pre-trial case management, compels parties to produce all information and documents as required by the court (Order 34, r.8 of RC 2012), and also introduced automatic directions for personal injury cases (Order 34, r.10 (1) of RC 2012), it excludes admiralty action and any action where the pleadings contain an allegation of a negligent act or omission in the course of medical or dental treatment (Order 34, r.10 (5) of RC 2012). Effectively this means that the difficulty in getting access to medical records is not overcome and the only recourse for discovering these documents is through civil action.

The current practice is for patients in medical suits to first bring claims against the doctor and then attempt to obtain the medical records. The plaintiffs have very little choice but to take a “shotgun” approach in drafting their allegations of negligence and this may be detrimental to their case, as the statement of claim must be supported by as much detail as the circumstances allow.

There is no legislation on data protection or access to health records to facilitate patients before commencing court action and it is important to reform the relevant Malaysian laws to allow pre-action discovery to any persons, whether they are likely to be potential defendants or not, before the issuance of a writ. There is need for strong and unambiguous legislation to allow access to health records in both the public and private sectors.

#### 2.1.4 The Expert Medical Witness

Another critical element in medical negligence actions is obtaining expert medical witness opinions on whether the particular act or omission concerned constituted a breach of duty. Generally, in the tort system the only acceptable proof of the standard of care is the testimony or the reasonable professional opinion of another doctor. This is often difficult to obtain as doctors may be unwilling to provide evidence against their colleagues. This situation creates among the legal profession a view of physicians as being “conspirators of silence.” Such a sentiment arises from the fact that physicians served with civil notice of a pending medical malpractice case are advised by their lawyers to maintain silence and not to discuss the case details with anyone (Seubert, 2007).

Plaintiffs also do not have access to the expertise and resources available to the defendant doctor such as a union that may rally to the doctor’s defense. This poses a problem because the experts are able to withhold materials which may be detrimental to their case. Under the current system, the first step after appointing a lawyer is for the patient to obtain the records for examination by a medical expert in order to prepare an initial report. The medical expert must be willing to give oral evidence and be cross-examined at the later part of proceedings (Kassim, 2008). However, in the event that “the case does not proceed to a contested hearing, the expert evidence on both sides is still likely to be instrumental in bargaining process leading to an out of court settlement, or even to a decision by claimant to abandon proceedings” (Philips, 1997).

Undoubtedly, there are a great number of obstacles faced by legal practitioners in obtaining expert evidence for claimants in medical negligence cases. Moreover, even if an expert witness is available to testify, there is no guarantee that the witness would be able to convince the court that negligence had been the proximate cause of the plaintiff’s injuries. It is left to the courts to decide on whether the evidence given by expert medical witness withstands logical analysis and be accepted.

#### 2.1.5 Accountability

The main consideration in pursuing medical negligence cases is to seek an explanation or an apology to ensure that there is no repetition. Paradoxically, the risks inherent in following lawsuits through and initiating litigation act as a deterrent to that intention. An article in the “American Academy of Orthopedic Surgeons” (AAOS) referred to a survey where money was fourth on the list behind disclosure, desire for apology, and prevention of future errors as grounds for wanting to sue for medical malpractice (Sohn & Jayasankar, 2011). Lord Woolf in his Access to Justice Final Report 1996(at: Para 18) said, “Patients besides their need for compensation have also other needs, which the tort system fails to cater for. Similar to an explanation of why their injury occurred, accountability or an apology from the responsible doctor and making sure that the mishap dose not occurs again in the future also figure prominently”.

Physicians and hospitals fear that an apology will be used against them as an admission of negligence and opening a dialogue about what happened may simply provide ammunition for the plaintiff’s attorney during trial proceedings. If medical practitioners meet the patient’s need for information willingly, if the patient is assisted in processing the information, and if the patient believes that the system has responded with improvements, they may not seek recourse to legal action. Medical complaints can often be resolved at an early stage by supportive communication with patients on where things are thought to have gone wrong.

Unfortunately the current tort system is unable to cater to remedies such as prevention of recurrence, apologies and avenues for a dialogue between the parties. According to a Kaiser Family Foundation survey (2004), 70% of patients who experienced medical errors are not informed by their doctors. Similarly, a national survey from Columbia University’s Institute on Medicine as a Profession (IMAP, 2007) found that nearly half of US doctors failed to report incompetent colleagues or medical errors. Even in New Zealand, where doctors are protected from the threat of liability, 61% of patients who experienced medical errors reported that their doctor or health care professional never informed them of the error (Bogdan, 2011).

### 2.2 Rising Medical Malpractice Claims

Based on available data, it is clear that there is a significant increase in the number of lawsuits and litigation against physician and health care medical negligence cases worldwide. The California Medical Association (1977) using hospital data in California found that 0.79% of admissions, or 1 in 125 patients, experienced adverse events due to negligence. The California study also revealed that an adverse event occurred in 4.76% of hospitalizations, or about 1 for every 21 admissions. In addition, of the total number of adverse events in California in 1974, 1 in 6 (17%) was deemed attributable to negligence. Such rates were present despite physician liability for injury under the rules of negligence. This belies the theoretical basis of Shavell’s (1980) argument that negligence rules would encourage physicians to take “due care” resulting in no medical malpractice (Danzon, 1991).

The 2010 Annual Report of the Medical Defence Union (MDU) notes a sharp increase in the number of complaints especially from public bodies. This increase was driven by claims against general practitioners which rose 20% over the previous year, the highest number of cases seen since Civil Justice Reforms were introduced in 1999. In the second half of 2010, the average length of such hearings was more than 7.5 days (The Medical Defence Union limited: Report & Accounts, 2010). Moreover the number of clinical negligence claims reported to the NHSLA in the past five years increased 52% from 5,697 in 2005/2006 to 8,655 in 2010/2011. In 2011, the NHSLA spent £729.1 million in medical negligence costs, a 12% increase over 2009/2010 (Penningtons Clinical Negligence, Annual Report 2012).

In Malaysia, generally it is estimated that litigation numbers may be around 20 to 30 cases annually and the amount of compensation awarded in Malaysia is far below that made by US or English courts (Elango, 2003). However, negligence claims and the size of awards in this country are clearly on the rise. The amount of compensation paid by the Malaysian government to medico-legal cases increased from RM219, 508 in 2000, to RM430, 502 in 2001 and RM951, 889 in 2002 (Kassim, 2009).

According to the 2010 Ministry of Health Annual Report, compensation for court cases rose from RM1.2 million in 2006 to RM5.7 million in 2010. Payment for potential medico-legal cases and settled out of court also increased from RM25, 000 to RM906, 365 over the same period. This means that the total compensation from 2006 to 2010 was RM12.9 million, with a noticeable increase in 2009 from RM2.9 million to RM6.6 million in 2010. In the 5-year period beginning 2005, a total of 113 negligence cases involving government health care providers, mainly doctors, were settled in and out of court, of which Obstetrics and Gynecology (O&G) cases accounted for 42 or 37%. During the same period, RM6.7 million was paid out through court orders and ex gratia, making an average of RM58, 000 per case. In 2011, the High Court in Johor awarded RM870, 000 to a couple as compensation for the irreversible injury suffered by their son due to negligence in handling his delivery process (Abdullah, 2011).

It is clear that the tort system makes it difficult for injured patients to even initiate claims much less receive any accountability or compensation at the end of the court process. Litigation has proved to be detrimental to the relationship between physicians and patients by creating antagonism between them and preventing the dissemination of honest explanations for any injury caused in the medical process. In addition the lengthy period and expenses involved in litigation deters many injured patients from making claims. The growing dissatisfaction with the tort system has resulted in medical malpractice reforms being proposed by various countries in the effort to improve on the current litigation system. Some reforms seek to maintain the tort liability system with some modifications, others suggest broader changes in the malpractice claims processes, and calls have also been made to completely replace tort with a new system of compensating victims of adverse outcomes. Regardless of choosing which kind of solution for the current tort system, it is obvious that maintaining the present tort system without addressing fundamental issues is clearly an inadequate approach which harms both the litigation parties and the health care system.

In fact, the law of medical negligence, concerns not just an injured patient and a defendant doctor in a dispute but political and economy decision-making by the Executive of a government can also be influenced by how the law develops (Mason & Laurie, 2011). This is because the developments of the law of medical negligence can impact on the number of medical claims and the size of compensation awarded against defendant doctors. When there is a continual increase in medical negligence claims and the amount of damages awarded, public confidence in the delivery of health care service may be undermined and the medical indemnity industry may be under pressure to increase indemnity premiums (Lee, 2012).

## 3. Conclusion

This study shows that the litigation system has not performed satisfactorily in providing victims of medical injuries with fair and adequate accountability and compensation. For many injured patients the lengthy period and expenses involved deter recourse to the courts. The unwillingness of expert witnesses to provide evidence against their colleague and non-accessibility to medical records in the pre-action stage is often serious obstacles in initiating claims against physicians in court. It is obvious that maintaining the current tort system without solving fundamental issues is absolutely an inadequate approach which harms the litigation parties, the health care system and government. What is needed is a system that ensures greater physician accountability delivers fair compensation to victims and reduces the costly nature and lengthy periods involved in the litigation processes. In recognizing the issues involved and the need for medical liability reform, academic researchers have shown extensive interest in examining tort difficulties and evaluating how well traditional tort reform laws have worked. The number of studies has doubled over the past years. However, there is still a need to study options that not just introduce and suggest solutions, but to examine the extent of these proposed options in resolving tort problems in order to develop a model for use by countries seeking to reform their medical malpractice compensation systems. The future research should concentrate on reform options including alternative dispute resolutions, health courts and enacting apology provisions to protect delivering accountability by physicians in case of occurrence medical malpractice.
